# Diagnosis and Treatment of Endometriosis and Endometriosis-Associated Infertility: Novel Approaches to an Old Problem

**DOI:** 10.3390/jcm11133914

**Published:** 2022-07-05

**Authors:** Antonio Simone Laganà, Amerigo Vitagliano, Vito Chiantera, Ettore Cicinelli

**Affiliations:** 1 Unit of Gynecologic Oncology, ARNAS “Civico-Di Cristina-Benfratelli”, Department of Health Promotion, Mother and Child Care, Internal Medicine and Medical Specialties (PROMISE), University of Palermo, 90127 Palermo, Italy; vito.chiantera@unipa.it; 2 Unit of Gynecology and Obstetrics, Department of Women and Children’s Health, University of Padua, 35100 Padua, Italy; amerigo.vitagliano@gmail.com; 3 Unit of Obstetrics and Gynecology, Department of Biomedical Sciences and Human Oncology, University of Bari “Aldo Moro”, 70124 Bari, Italy; ettore.cicinelli@uniba.it

Endometriosis, defined by the presence of endometrial-like tissue, glad and stroma outside the uterus [1], is a polymorphous, subtle pathology that affects more than 1 in 10 women worldwide. This condition may severely affect quality of life, modifying well-being and relationships due to chronic pelvic pain, infertility and obstetrical complications [2].

Despite great advances in diagnostics, pharmacology, minimally invasive surgery and assisted reproduction technologies, endometriosis still represents an unsolved global health issue. There is an urgent need to improve our knowledge on endometriosis, as well as its clinical management.

In this context, this Special Issue aims to summarize cutting-edge developments within this topic in order to offer new insight and identify research priorities in the field. Among the articles published in the Special Issue, Scioscia et al. [3] aim to establish a common terminology between imaging diagnostics and modern surgical anatomy. Indeed, accumulating evidence suggests that ultrasound is an effective tool to detect and characterize lesions on the uterosacral ligament, parametrium, and paracervix [4]; this point is of paramount importance, considering that endometriosis may infiltrate the parametrium and may also involve the ureter, resulting in more complex surgery. In particular, endometriosis can involve the cervical section of the uterosacral ligament, which is close to important tissues, namely, the parametrium and paracervix, which contain vessels and important nerves and nerve anastomoses of the inferior hypogastric plexus. These efferent fibers are essential for bladder and rectal functionality, so tailored nerve-sparing surgery has become a standard approach for treating deep-infiltrating endometriosis [5].

Nevertheless, sometimes even surgery is not enough for the treatment of endometriosis, and thus medical approaches for the management of severe symptomatology are lacking. From this perspective, Donnez and Dolmans [6] perform an accurate systematic review aiming to evaluate the effectiveness of the most frequently applied medical options, namely oral contraceptive pills (OCPs) and progestogens, for the treatment of premenopausal women with endometriosis-associated pelvic pain, dysmenorrhea, non-menstrual pelvic pain and dyspareunia. Interestingly, they found that OCPs and progestogens are effective only in two-thirds of women suffering from endometriosis, suggesting that other options, such as oral gonadotropin-releasing hormone antagonists [7], may be adopted in cases of failure or intolerance to first-line medical treatment (as occurring in one-third of women due to progesterone resistance).

Finally, Aimagambetova and collaborators [8] summarized the most robust pieces of evidence regarding fertility-sparing approaches for pre-menopausal nulliparous women affected by early-stage endometrioid endometrial cancer. Based on a comprehensive overview, they confirm that minimally invasive surgical techniques combined with progestogens, namely, medroxyprogesterone acetate or megestrol acetate, may be considered as valid options in cases without myometrial involvement and lymph-vascular space invasion [9,10].

Considering the high quality of the articles submitted and published in this Special Issue, we would like to thank all the authors for their precious contributions which will pave the way for new investigations in the field of endometriosis and endometriosis-associated infertility. Further efforts of the scientific community, in conjunction with the valuable contributions and suggestions made by the authors of this Special Issue, are necessary to sharpen weapons against a pathology that is still a bleeding wound of gynecology, with a huge number of social victims desperately waiting for medical help.
